# Pulmonary alveolar regeneration in adult COVID-19 patients

**DOI:** 10.1038/s41422-020-0369-7

**Published:** 2020-07-06

**Authors:** Jingyu Chen, Huijuan Wu, Yuanyuan Yu, Nan Tang

**Affiliations:** 1 Wuxi Lung Transplantation Center, Wuxi People’s Hospital affiliated to Nanjing Medical University, Wuxi, Jiangsu 214023 China; 2 School of Life Sciences, Tsinghua University, Beijing, 100084 China; 3 National Institute of Biological Sciences, Beijing, 102206 China; 4 Tsinghua Institute of Multidisciplinary Biomedical Research, Tsinghua University, Beijing, 100084 China

**Keywords:** Adult stem cells, Regeneration

Dear Editor,

Alveolar regeneration after an acute lung injury has been observed in many mammals. Results in animal models have shown that alveolar type II (AT2) cells function as resident alveolar stem cells that can proliferate and differentiate into alveolar type I (AT1) cells to build new alveoli after lung injury.^1^ However, alveolar regeneration after acute lung injury in adult humans is still poorly characterized, mainly due to the lack of lung samples and regeneration-specific molecular markers. In patients with COVID-19 pneumonia, the severe acute respiratory syndrome coronavirus 2 (SARS-CoV-2) can directly attack alveolar epithelial cells and cause massive AT2 cell death. It is unknown whether alveolar regeneration occurs upon SARS-CoV-2 infection-induced lung injury. This knowledge will substantially improve our basic understanding of the COVID-19 disease and our ability to prognosticate patient outcomes.

In this study, we enrolled two COVID-19 patients. Patient-1 is a 58-year-old male and patient-2 is a 54-year-old male. Prior to the SARS-CoV-2 infection, both patients did not have signs of lung disorders. In the course of the disease, noninvasive ventilation, intubation, invasive ventilation, and extracorporeal membrane oxygenation (ECMO) were used in succession (Supplementary information, Fig. S1a, b).^2^ An emergency lung transplant was performed on patient-1 on disease onset day 38 due to hemorrhage in his lungs (Supplementary information, Fig. S1a). The patient-2 received lung transplants on disease onset day 90 due to extensive pulmonary fibrosis (Supplementary information, Fig. S1b).

Hematoxylin and eosin (H&E) staining of the lung specimen from both patients revealed that multiple cell aggregates were still evident in the alveolar lumen, indicating severe diffuse alveolar damage (Fig. 1a). Immunostaining showed signs of lung fibrotic changes, including significant collagen I deposition and proliferating α-SMA^+^ myofibroblasts (Supplementary information, Fig. S2a, b). In most regions of the lungs of both patients, very few HTI-56^+^ AT1 cells were observed, indicating a significant depletion of AT1 cells (Fig. 1b). Notably, we observed that alveolar regions harbored a large number of clustered AT2 cells lining the alveolar epithelium in patients’ lungs (Fig. 1b). About 1.1% of AT2 cells in the lung of patient-1 and 10% of AT2 cells in the lung of patient-2 stained positive for Ki67, indicating that AT2 cells are proliferating (Fig. 1b). Both phosphohistone H3 and PCNA staining results also indicate the increased proliferation of AT2 cells (Supplementary information, Fig. S3a, b). Together, these findings establish that AT2 cells are able to replicate additional AT2 cells after SARS-CoV-2-induced lung injury.

**Fig. 1:**
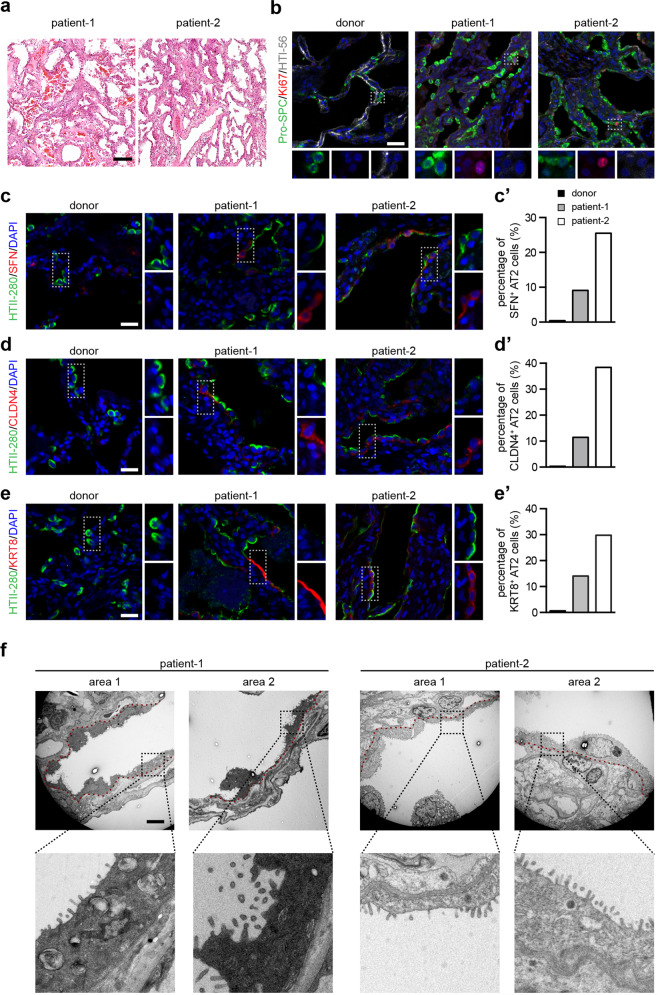
The proliferation and differentiation of the resident alveolar stem cells in the lungs of two COVID-19 patients. **a** H&E staining of the lungs of two COVID-19 patients. **b** Immunostaining using antibodies against proSPC, Ki67, and HTI-56 of a healthy donor lung and lungs of two COVID-19 patients. **c**–**e′** Immunostaining using antibodies against HTII-280 and SFN (**c**), HTII-280 and CLDN4 (**d**), and HTII-280 and KRT8 (**e**) of a healthy donor lung and the COVID-19 lungs. The percentage of intermediate AT2 cells in total AT2 cells were quantified (**c′**, **d′**, and **e′**). **f** TEM images of the COVID-19 lungs. The red dashed line indicates the location of the basement membrane. Scale bars, 100 µm (**a**), 20 µm (**b**–**e**), 5 µm (**f**).

Previous studies in both human and mouse lungs have confirmed the existence of a transient intermediate cell state for AT2 cells which occurs during differentiation of AT2 cells into AT1 cells.^3–7^ AT2 cells in this intermediate cell state subsequently differentiate into AT1 cells. Three markers with transient expression profiles, Claudin4 (CLDN4), Stratifin (SFN), and Keratin 8 (KRT8), are known to specifically define this intermediate AT2 cell state. Few AT2 cells in the healthy donor lung expressed any of these markers. In contrast, many HTII-280^+^ AT2 cells in the COVID-19 lungs expressed CLDN4, SFN, or KRT8 (Fig. 1c–e). Some AT2 cells that express CLDN4, SFN, or KRT8 show decreased expression levels of HTII-280 and proSPC (Supplementary information, Fig. S3c). Collectively, these observations confirm that AT2 differentiation was initiated in the lungs of COVID-19 patients.

Given the knowledge that markers of AT2 intermediate cell state cell are transiently expressed in differentiating AT2 cells but not expressed in AT1 cells, we next used transmission electron microscopy (TEM) to investigate the potential differentiation of AT2 cells (Fig. 1f). Consistent with our immunostaining results that show a significant depletion of AT1 cells in the COVID-19 lungs (Fig. 1b), few AT1 cells were observed by the TEM analysis. However, the TEM images showed that multiple regions typically occupied by AT1 cells in normal lungs actually harbored numerous squamous alveolar epithelial cells (Fig. 1f). Although these cells did contain some AT2-cell-like features—including lamella bodies and distinct apical microvilli—they exhibited the characteristic flattened and elongated shape of AT1 cells (Fig. 1f). The expression of the intermediate AT2 cell markers can be also detected in AT2 cells of idiopathic pulmonary fibrosis (IPF) lungs,^4,5,7^ however, no squamous and elongated AT2 cells can be observed in IPF lungs by TEM analysis, supporting the hypothesis that AT2 cells in IPF patients may be impaired in further differentiation into AT1 cells.^3,7^ Together, these findings indicate that AT2 cells of the lungs of COVID-19 patients can apparently participate in alveolar regeneration by differentiating into AT1-like cells. Additionally, our results establish that alveolar regeneration can be activated by humans in their 50s.

An interesting remaining question concerns the time scale of the alveolar regeneration process in adult human lungs. In rodents, it takes a few weeks to regenerate the alveoli and restore lung functions. To date, there is no experimental evidence that new alveolar structures can be created in the adult human lung. One medical study showed that a 33-year-old woman who had undergone pneumonectomy had alveolar growth over a period of 15 years; this study relied on MRI-based assessment of lung microstructure and analyses of lung function.^8^ Our results demonstrate that alveolar regeneration in COVID-19 lungs was initiated by (at the latest) the 38th day after the onset of initial symptoms. These observations indicate that it may take a few weeks for AT2 cells to repopulate and to differentiate into AT1 cells in adult humans after acute lung injuries. Future imaging studies that analyze the lung microstructure of patients should provide further insights into whether new functional alveolar structures will form in COVID-19 patients.

Pulmonary fibrosis is a frequent complication in patients with viral pneumonia-induced acute respiratory distress syndrome. However, CT scans have shown that the signs of pulmonary fibrosis after viral pneumonia can partially regress over time.^9^ It seems plausible that alveolar regeneration may also occur in patients with less severe viral pneumonia, and to speculate that such regeneration functions to help to restore lung function and even resolve pulmonary fibrosis. Our observations open the door for future studies to elucidate the mechanisms that regulate human alveolar regeneration after acute lung injury and facilitate to prognosticate the outcomes of COVID-19 patients.

## Supplementary information

Supplementary information, Figures and Methods
