# Supplementary material for: Pulmonary alveolar regeneration in adult COVID-19 patients
Source: Cell Res. 2020 Jul 6;30(8):708–10. doi: 10.1038/s41422-020-0369-7 (PMC7338112; doi:10.1038/s41422-020-0369-7)
Supplement: Supplementary file 1 — Supplementary information, Figures and Methods [file 41422_2020_369_MOESM1_ESM.pdf]

Supplementary information, Fig. S1

a

	Days after illness onset	1	2	3	4	5	6	7	8-16	17-24	25-30	31 – 35	36	37	38
COVID-19 patient-1	SARS-CoV-RNA					+		+			–	–			
	mechanical ventilation														
	ECMO														
	lung hemorrhage														
	lung transplantation														

b

	Days after illness onset	1	2	3	4	5	6-16	17 – 52	53-89	90
COVID-19 patient-2	SARS-CoV-RNA					+			–	
	mechanical ventilation									
	ECMO									
	lung transplantation									

Supplementary information, Fig. S1. The medical history of the two COVID-19 patients.

(a) A summary of the medical history of the COVID-19 patient 1.

(b) A summary of the medical history of the COVID-19 patient 2.

Supplementary information, Fig. S2

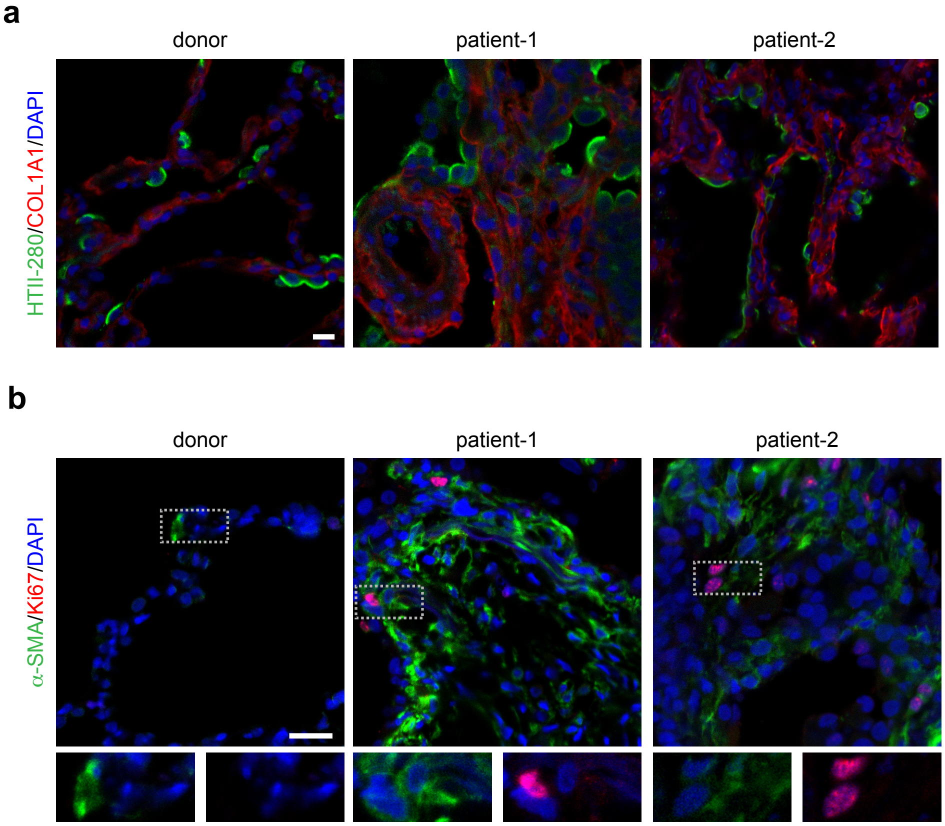

Supplementary information, Fig. S2. The COVID-19 lungs show fibrotic changes.

(a) Immunostaining using antibodies against HTII-280 and COL1A1 of a healthy donor lung and lungs of two COVID-19 patients.

(b) Immunostaining using antibodies against α-SMA and Ki67 of a healthy donor lung and lungs of two COVID-19 patients.

Scale bars, 20μm (a, b).

Supplementary information, Fig. S3

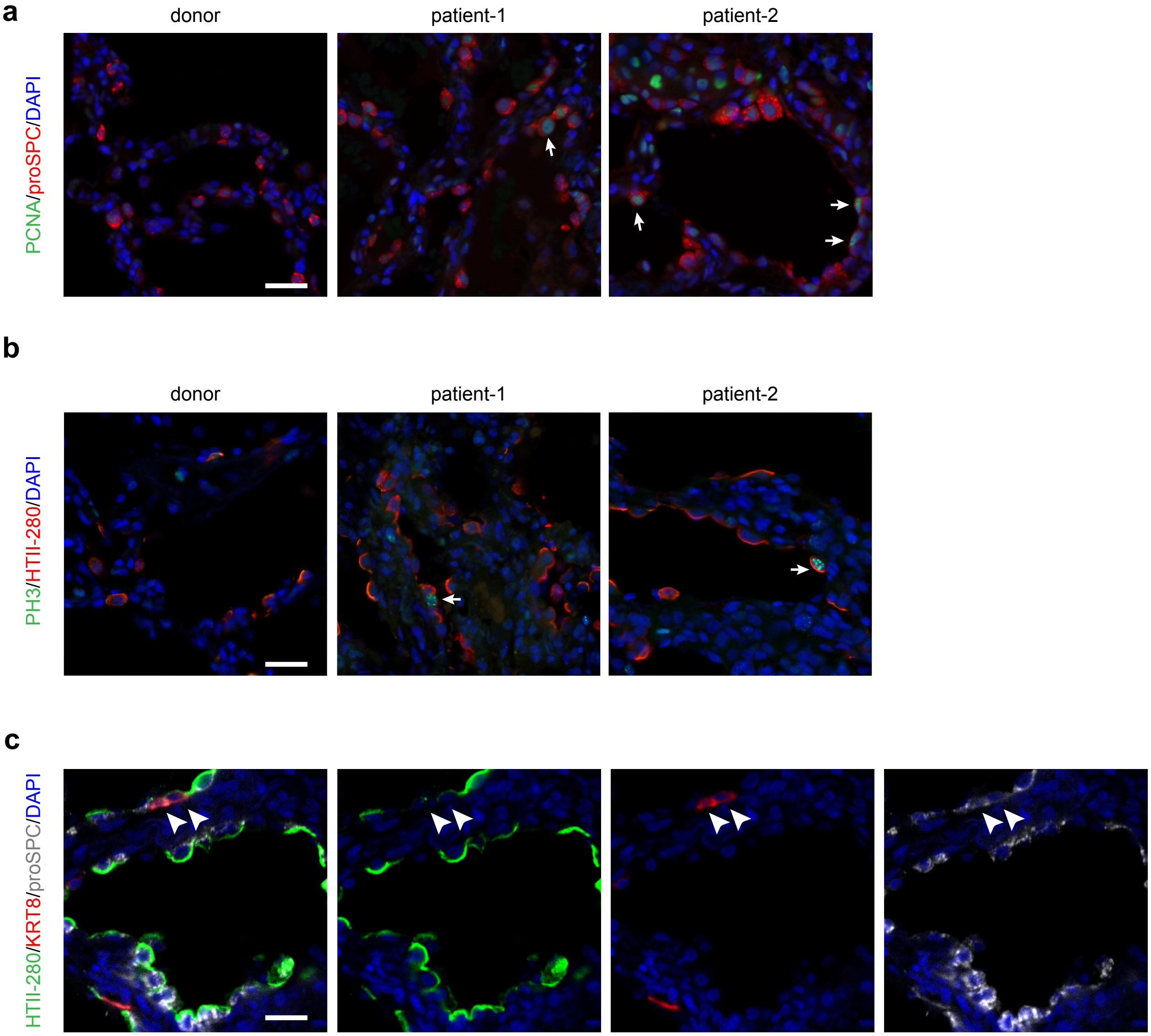

Supplementary information, Fig. S3. More characterization of AT2 cells in lungs of COVID-19 patients.

(a) Immunostaining using antibodies against PCNA (green) and proSPC (red) of a healthy donor lung and lungs of two COVID-19 patients. White arrows indicate PCNA positive AT2 cells.

(b) Immunostaining using antibodies against phosphohistone H3 (PH3) (green) and HTII-280 (red) of a healthy donor lung and lungs of two COVID-19 patients. White arrows indicate PH3 positive AT2 cells.

(c) Some AT2 cells at the intermediate cell state show reduced levels of AT2 markers.Some KRT8 expressing AT2 cells (arrowhead) show reduced expression levels of HTII-280 (green) or proSPC (grey).

Scale bars, 20μm.

MATERIALS AND METHODS

Human specimens

All experiments with human tissue samples were performed under protocols approved by the Institutional Review Boards at National Institute of Biological Sciences, Beijing and Wuxi People's Hospital. The COVID-19 lung tissues were collected from lung explants of two COVID-19 patients. The patient-1 is a 58-year-old male and the patient-2 is a 54-year-old male. The healthy donor lung tissues (male, age 56yr) were resected on the basis of size incompatibility.

Immunostaining experiments

The human lung tissues were fixed with 10% neutral buffered formalin, cryoprotected in 30% sucrose, and embedded in OCT. 15μm thickness cryosections were used for immunostaining experiments. The following primary antibodies were used for immunostaining experiments: anti-COL1A1 (rabbit, 1:300, Abcam, ab34710), anti-α-SMA (mouse, 1:300, Sigma, C6198), anti-proSPC (rabbit, 1:500, Millipore, AB3786), anti-HTII-280 (mouse, 1:50, Terrace Biotech, TB-27AHT2-280), anti-HTI-56 (mouse, 1:50, Terrace Biotech, TB-29AHT1-56), anti-Ki67 (rat, 1:200, Invitrogen, 14-5698-82), anti-PCNA (mouse, 1:200, Santa Cruz, sc-56), anti-PH3 (rabbit, 1:200, Millipore, 07-690), anti-CLDN4 (rabbit, 1:500, Invitrogen, 36-4800), anti-SFN (rabbit, 1:500, Invitrogen, PA5-95056), anti-KRT8 (rat. 1:200, DSHB, TROMA -I). All of the Alexa Fluor coupled secondary antibodies were used at 1:500 dilutions. For the KRT8, CLDN4, and SFN immunostaining experiments, the tyramide (Perkin Elmer, NEL744001KT) signal amplification method was used followed the manufacturer's recommendations.

Hematoxylin and Eosin (H&E) staining

Lungs were fixed with 10% neutral buffered formalin, cryoprotected in 30% sucrose and embedded in OCT, finally cut into 15μm thick cryosections. The Hematoxylin (Abcam, ab150678) & Eosin (Sigma, HT110280) staining experiment followed the standard H&E protocol.

Transmission electron microscope

The human lung tissues were fixed with a mixture of 4% PFA and 0.4% glutaraldehyde for 24 hours at 4°C. Lungs were cut into small pieces (about $1mm^3$) and soaked in 1% antimony tetraoxide for 1 hour at 4°C. Then the tissues were dehydrated in acetone, and embedded by resin. The embedded resin blocks were cut into ultra-thin sections and further stained using uranium acetate and lead citrate. The TEM pictures were taken by a transmission electron microscopy (FEI, Tecnai spirit G2).

QUANTIFICATION

Lung tissues were collected from 3 different regions in the lungs of COVID19 patients. The proliferation rate of AT2 cells and the proportion of AT2 cells expressing the intermediate AT2 markers were quantified as the average of three different lung regions.
